# Evaluation of dose prediction error and optimization convergence error in four‐dimensional inverse planning of robotic stereotactic lung radiotherapy

**DOI:** 10.1120/jacmp.v14i4.4270

**Published:** 2013-07-08

**Authors:** Mark K.H. Chan, Dora L.W. Kwong, Anthony Tong, Eric Tam, Sherry C.Y. Ng

**Affiliations:** ^1^ Department of Clinical Oncology The University of Hong Kong Hong Kong China; ^2^ Department of Clinical Oncology Tuen Mun Hospital Hong Kong China; ^3^ Department of Clinical Oncology Queen Mary Hospital Hong Kong China; ^4^ Theresa Po CyberKnife Center Hong Kong China

**Keywords:** 4D optimization, Monte Carlo dose calculation, CyberKnife, SBRT

## Abstract

Inverse optimization of robotic stereotactic lung radiotherapy is typically performed using relatively simple dose calculation algorithm on a single instance of breathing geometry. Variations of patient geometry and tissue density during respiration could reduce the dose accuracy of these 3D optimized plans. To quantify the potential benefits of direct four‐dimensional (4D) optimization in robotic lung radiosurgery, 4D optimizations using 1) ray‐tracing algorithm with equivalent path‐length heterogeneity correction (${4\text{EPL}}^{\text{opt}}$), and 2) Monte Carlo (MC) algorithm (${4\text{MC}}^{\text{opt}}$), were performed in 25 patients. The ${4\text{EPL}}^{\text{opt}}$ plans were recalculated using MC algorithm (${4\text{MC}}^{\text{recal}}$) to quantify the dose prediction errors (DPEs). Optimization convergence errors (OCEs) were evaluated by comparing the ${4\text{MC}}^{\text{recal}}$ and ${4\text{MC}}^{\text{opt}}$ dose results. The results were analyzed by dose‐volume histogram indices for selected organs. Statistical equivalence tests were performed to determine the clinical significance of the DPEs and OCEs, compared with a 3% tolerance. Statistical equivalence tests indicated that the DPE and the OCE are significant predominately in ${\text{GTV D}}_{98\%}$. The DPEs in $V_{20}$ of lung, and $D_{2\%}$ of cord, trachea, and esophagus are within 1.2%, while the OCEs are within 10.4% in lung $V_{20}$ and within 3.5% in trachea $D_{2\%}$. The marked DPE and OCE suggest that 4D MC optimization is important to improve the dosimetric accuracy in robotic‐based stereotactic body radiotherapy, despite the longer computation time.

PACS numbers: 87.53.Ly, 87.55.km

## I. INTRODUCTION

Inverse optimization of intensity‐modulated stereotactic body radiotherapy (IM‐SBRT) is typically performed using conventional ray‐tracing or pencil beam (PB) convolution algorithms with radiological equivalent path length (EPL) for tissue heterogeneity during the optimization stage and in the final dose calculation.^1^, ^2^, ^3^, ^4^ Numerous studies have demonstrated the deficiency of these algorithms to predict the tumor dose distribution in low‐density regions such as thorax, with the largest discrepancies observed at the tissue–lung interfaces due to serious lateral electronic disequilibrium.^1^, ^5^ It can be expected that when the set of “optimized” IM‐parameter (e.g., beamlets weight and beam orientation) derived from conventional dose calculation algorithms is applied to obtain the final dose distribution using a gold standard method (e.g., Monte Carlo algorithm), the resultant dose distribution and relevant target dose constraints will be nowhere close to the initially optimized ones. The difference between the PB/ray‐tracing algorithms and the MC algorithm contributes to systematic dose prediction error (DPE). When these algorithms are employed during inverse optimization, optimization convergence error (OCE) occurs because the DPE causes the optimizer to converge to suboptimal solution.^(6)^

For lung SBRT, inverse optimization is further complicated concerning the temporal changes of patient anatomy. As the patient breathes, the lung expands and compresses resulting in variation of density within each lung voxel to conserve lung mass. Jin et al.^(7)^ have investigated the relationship between beam margin and the lung density. They suggested that either the beam margin or the prescription level had to be varied in order to maintain the desired dose coverage with the lung density changing. The fact that dose optimization is typically done on a single CT scan and uses a constant margin implies the prescription dose level is not guaranteed optimized in terms of target dose coverage and other normal tissue constraints throughout the breathing cycle.

Four‐dimensional (4D) dose calculation is a powerful tool to explicitly include the effect of organ motion. The general approach of 4D dose calculation is to employ deformable vector fields obtained from deformable image registrations to warp dose distributions calculated on different breathing geometries to a reference geometry so that the cumulative effect of organ motion can be accounted for. So far, 4D dose calculation has been predominately performed outside the commercial treatment planning systems, and is mostly employed as a tool for assessing, rather than including, the cumulative effect of organ motion and deformation. Starkschall et al.^(8)^ applied standard 3D plans created on one single breathing instance to other breathing instances and deformably summed these independent dose distributions to compose the 4D dose distribution. They showed that 4D dose calculation has the potential to detect unexpected decrease of tumor dose and increase of organs‐at‐risk (OAR) doses. Four‐dimensional dose calculation frameworks of similar kind are valuable in terms of evaluating the dosimetric impacts of organ motion, but do not help to avoid violations of dose constraints as specified in the plans that are optimized on the static 3D CT images. A few investigators have taken the logical step to extend the capability of 4D dose calculation to build different frameworks of 4D dose optimization in conventional linac‐based intensity‐modulated radiotherapy (IMRT).^9^, ^10^, ^11^ Despite the theoretical advantages of 4D MC optimization (4D MCO), MultiPlan of CyberKnife remains the sole commercial treatment planning system (TPS) that supports direct 4D optimization in SBRT. To date, no results have been reported the potential benefits of 4D MCO in real‐time tumor tracking SBRT using CyberKnife. In fact, 4D MCO is particularly useful to include this dynamic movement of treatment beam while taking into account of the dosimetric effects of breathing‐induced geometry and density changes during plan optimization. The aim of this work is to investigate the potential advantages of direct four‐dimensional (4D) MC optimization (4D MCO) to improve dose accuracy in robotic‐based IM‐SBRT.

## II. MATERIALS AND METHODS

### A. Patient selection and treatment planning

This study included a cohort of 25 lung cancer patients previously treated with CyberKnife (Accuray Inc., Sunnyvale, CA) (Table 1). Each patient had a four‐dimensional computed tomography (4D CT) scan. The 4D CT acquisition and reconstruction protocols were described in our previous study.^(12)^ Each 4D CT study was reconstructed into ten equal time binned 3D CT images. All treatments were performed under real‐time tumor tracking using either the Synchrony Respiratory Tracking System (RTS) (Accuray Inc.) or XSight Lung Tracking System (Accuray Inc.). Technical descriptions of these tracking methods were previously described by Kilby et al.^(13)^ The lung tumors ranged in different sizes and locations. Characteristics of these tumors were described in Table 1. In this study, plans were designed to deliver either a total dose of 60 Gy in three fractions or 48 Gy in four fractions to 95% of the planning target volume (PTV), which was expanded by 5 mm from the gross tumor volume (GTV). Other planning objectives included sparing of normal lung tissue, cord, trachea, and esophagus.

**Table 1:** Tumor characteristics

Total Patient Number	25
Gross Tumor Volume (cc)	
Median	10.2
Range	0.6–77.4
Planning Tumor Volume (cc)	
Median	27.8
Range	4.2–134.5
Principal Motion Amplitude (mm)	
Median	7.0
Range	1.0–22.5
Tumor Location	
Upper lobe	4 (16%)
Middle lobe	17 (68%)
Lower lobe	7 (28%)

### B. Dose calculation algorithms

The ray‐tracing and Monte Carlo patient dose calculation algorithms available in the CyberKnife TPS MultiPlan v.4.0.x (Accuray Inc.) were used for the inverse optimization. These algorithms have been previously described and compared by different authors.^1^, ^14^, ^15^ Briefly, the ray‐tracing algorithm calculates dose by applying heterogeneity correction factors to scale the tissue phantom ratios of the measured broad beam data along beam central axis. Therefore, dose discrepancies may result from the assumption of charged particle equilibrium. The Monte Carlo dose engine is based on a single source model that describes the source distribution, energy distribution, and fluence distribution.^(16)^ The MultiPlan TPS uses a piecewise algorithm to convert the CT numbers to mass density and material. Each voxel in the patient geometry is assigned a mass density ρ for scaling the mean free paths (MFPs) of photons, and the steps of electron and positron tracks through each voxel. A material type is also assigned to each voxel (air ifρ<0.1g/cc, soft tissue if 0.1≤ρ≤1.125, or bone if >1.125) for defining the photon MPFs with each material type as a function of photon energy. The MC algorithm uses various variance reduction techniques (VRTs) including photon interaction forcing, photon splitting, employing Russian roulette, electron history repeating, and electron range rejection for efficient particle transports in the 3D rectilinear voxel geometry of the patient model. The VRTs implemented within this MC algorithm utilize a voxel model that considers various materials as being equivalent to variable density water for the purpose of the electron transport calculations. Therefore, the resulting dose quantity from the electron transport algorithm is expressed in terms of absorbed dose to variable density water, which is virtually identical to conventional MC‐calculated absorbed doses to media for most biological materials including lung and cortical bone.^(17)^

### C. 4D planning framework and 4D optimization schemes

**Figure 1:**
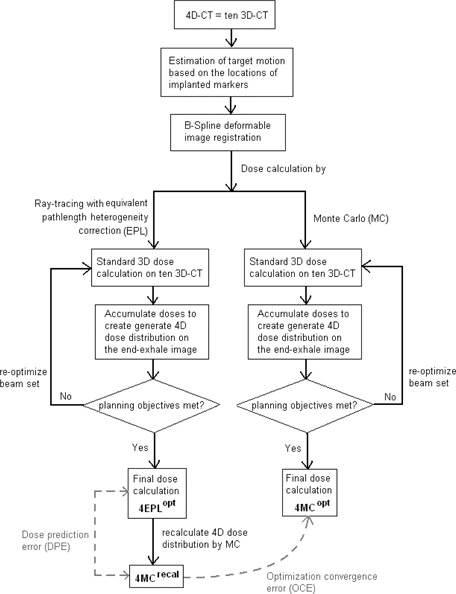
Illustrations of the 4D planning architecture and the 4D optimization schemes using equivalent path‐length (EPL) dose calculation algorithm and using Monte Carlo dose calculation algorithm. The dashed lines indicated the dose prediction error, quantified by the difference between ${4\text{EPL}}^{\text{opt}}$ and ${4\text{MC}}^{\text{recal}}$ methods, and the optimization convergence error (OCE), quantified by difference between ${4\text{MC}}^{\text{recal}}$ and ${4\text{MC}}^{\text{opt}}$.

Direct 4D inverse optimization was carried out with the 4D planning module in the MultiPlan TPS. The 4D planning architecture is illustrated in Fig. 1. As input, 4D CT images were needed for the construction of a patient breathing model. One of the core components of the 4D planning system is the dose mapping method that relates the dose deposition of each anatomic voxel between multiple samples of breathing geometry. Due to the notable tissue expansion and compression during respiration, a one‐to‐one correspondence between voxels of different breathing geometry generally does not exist. The MultiPlan TPS generated a series of voxel correspondence maps or deformation vector fields (DVFs) using a B‐spline deformation image registration (DIR) algorithm.^(18)^ In the 4D planning module, image registration was carried in a two steps.^(18)^ First, rigid registrations were performed to estimate locations of the target at different time point over the breathing cycle and served as the basis for shifting the beam aperture to reflect the target motion.^(19)^ In the second step, a dual resolution hybrid point and intensity‐based approach of deformable image registrations was used to produce deformation vector fields (DVFs) that were calculated between adjacent images in the 4D CT study. Results of the evaluation of this DIR algorithm were referred to Stancanello et al.^(20)^ The resultant DVFs were then applied to accumulate the dose distributions calculated at different respiratory states back onto the reference dose grid of the end‐of‐exhale (EOE) state to compose the 4D dose distribution. During optimization, the 4D dose matrix was continuously updated at each optimization step and displayed on the reference CT frame in which all structure contours were defined.

In this study, two 4D optimizations were run for each 4D CT scan (Fig. 1), first with the conventional ray‐tracing algorithm with equivalent path length heterogeneity correction (${4\text{EPL}}^{\text{opt}}$), and then restarted with the Monte Carlo dose engine (${4\text{MC}}^{\text{opt}}$). All optimizations were carried out using the sequential optimization algorithm. The sequential algorithm was executed in a series of optimization steps, each of which performs a simplex optimization applied to a single objective cost function designed to correspond to a specific clinical objective (e.g., target dose conformity, target dose coverage, maximum/mean dose to critical organs).^(21)^ The initial number of beams available to solution space depended on the number of predefined circular aperture(s) (approximately 2000 and 3000 beams for two and three circular collimators, respectively). To eliminate biases introduced by the planner, both optimizations were carried out with the same set of planning objectives that were determined from a large number of test cases. Considering that a significant amount time was needed to calculate the dose distributions on different patient geometries and to update the 4D dose matrix upon dose accumulation on the reference grid, the 4D MCO was performed in a medium resolution grid ($∼2.1\times 2.1\times 1.25\;{\text{mm}}^{3}$) and with 4% statistical uncertainty σ at the maximum dose point ($D_{\text{max}}$). For evaluations of the dose prediction and optimization convergence errors, all ${4\text{EPL}}^{\text{opt}}$ plans were recalculated with the MC dose engine, yielding the ${4\text{MC}}^{\text{recal}}$ plans. All final MC dose distributions were calculated using high resolution grid ($∼1.05\times 1.05\times 1.25\;{\text{mm}}^{3}$) and at 1% statistical uncertainty. For the present work, the time required to come up with a reasonable ${4\text{MC}}^{\text{opt}}$ plan varied from seven to more than 12 hours on the current system (Intel Xeon Processor 3.00 GHz), depending on the number of apertures and treatment beams in question. Note that this excluded the time spent on structure contouring and dose distribution fine‐tuning.

### E. dosimetric evaluations

Dosimetric evaluations were performed by analyzing the dose‐volume histograms (DVHs). The DVH indices of given tissues $D_{V}(\text{Gy})$ and $V_{D}(\%)$ are equal to the dose level D (Gy) covering volume V (%), and the volume V (%) covered by a given dose level D (Gy), respectively. The evaluated DVH indices included ${\text{GTV D}}_{98\%},{\text{cord D}}_{2\%},{\text{esophagus D}}_{2\%},{\text{trachea D}}_{2\%}$, and ${\text{lung V}}_{20}\;\text{Gy}$. The $D_{98\%}$ and $D_{2\%}$ indices are recommended as surrogates of the minimum dose and maximum dose in the ICRU Report 83.^(22)^ In addition, the $D_{98\%}$ and $D_{2\%}$ are less susceptible to statistical fluctuations in MC dose calculations, as suggested by Sakthi et al.^(23)^

As previously mentioned in the Introduction, the dose prediction error (DPE) due to EPL algorithm will be defined as the difference of dose result between the ${4\text{EPL}}^{\text{opt}}$ plan and the ${4\text{MC}}^{\text{recal}}$ plan that was obtained by recalculating the ${4\text{EPL}}^{\text{opt}}$ plan with the MC dose engine (Fig. 1). The DPE reflects differences introduced by variations of patient tissue heterogeneity during respiration. Considering the MC algorithm as the gold standard, the DEP is quantified by comparing DVH indices as:

(1) $$DPE=D_{v}^{4MC^{recal}}-D_{v}^{4EPL^{opt}}$$

or

(2) $$DPE=V_{D}^{4MC^{recal}}-V_{D}^{4EPL^{opt}}$$

On the other hand, the optimization convergence error (OCE) due to the EPL algorithm is defined as the difference of dose result between plans optimized with the MC algorithm (i.e., ${4\text{MC}}^{\text{opt}}$) and the EPL algorithm (${4\text{EPL}}^{\text{opt}}$). The OCE source addressed by this work is the accuracy of the dose calculations employed during the optimization process (Fig. 1). Similar to Eq. (1) and Eq. (2), the OCE is quantified by comparing DVH indices as:

(3) $$OCE=D_{v}^{4MC^{opt}}-D_{v}^{4MC^{recal}}$$

or

(4) $$OCE=V_{D}^{4MC^{opt}}-V_{D}^{4MC^{recal}}$$

In evaluating the DPEs and OCEs, DVH indices from the ${4\text{MC}}^{\text{recal}}$ and ${4\text{MC}}^{\text{opt}}$ solutions were taken as references, respectively. As pointed out in the AAPM Report 65, a dose calculation accuracy of 2%–3% is required to achieve an overall uncertainty of ≅5%.^(24)^ Therefore, a 3% difference from the reference DVH indices was considered clinical significant. Furthermore, the equivalent test method was adopted to determine the minimum $D_{V}(\text{Gy})$ or $V_{D}(\%)$ intervals around the reference ${4\text{MC}}^{\text{recal}}$ and ${4\text{MC}}^{\text{opt}}$ values such that *t*‐tests concluded that the reference and test $D_{V}(\text{Gy})$ or $V_{D}(\%)$ values in the DPE and OCE evaluations were equivalent with p<0.05.^(25)^ In brief, the $D_{V}$ or $V_{D}$ intervals around the reference ${4\text{MC}}^{\text{recal}}$ and ${4\text{MC}}^{\text{opt}}$ values were initially set to zero. They were then increased in 0.01 Gy steps until results of the paired *t*‐tests indicated equivalence between the ${4\text{EPL}}^{\text{opt}}$ and ${4\text{MC}}^{\text{recal}}$ solutions, and between the ${4\text{MC}}^{\text{recal}}$ and ${4\text{MC}}^{\text{opt}}$ solutions with p‐values >0.05 in the DPE and OCE evaluations, respectively. The percentage of the $D_{V}$ or $V_{D}$ intervals with respect to the averaged reference DVH values of each structure were then compared with 3% to determine if the $D_{V}$ or $V_{D}$ intervals of the DPE were clinically significant. Similarly, the equivalent test was performed to determine the clinical significance of OCE for each structure.

## III. RESULTS

**Figure 2:**
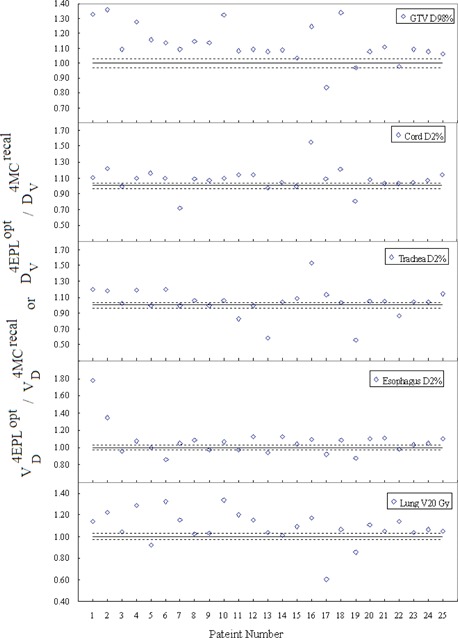
Normalized DVH indices resulting from comparison of ${4\text{EPL}}^{\text{opt}}$ with ${4\text{MC}}^{\text{recal}}$ for different organs. The differences quantify the magnitude of DPEs. The dotted lines indicate the 3% tolerance and the solid line indicates the baseline when the dose prediction errors are nil. The patient number was ordered with ascending GTV size.

Figure 2 shows the DPEs of different DVH indices for each patient. Note that the patient number was ordered in ascending GTV size. The results were normalized to $D_{v}^{4{\text{MC}}^{\text{recal}}}$ or $V_{D}^{4{\text{MC}}^{\text{recal}}}$ to facilitate intercomparisons between patients receiving different prescription oses. Notable differences occur in the ${\text{GTV D}}_{98\%}$, indicating that the ${4\text{EPL}}^{\text{opt}}$ method overestimated the ${\text{GTV D}}_{98\%}$ by a factor of 1.13, on average, and up to 1.36 in one patient. The DPE in ${\text{GTV D}}_{98\%}$ increases with decreasingly GTV size (Spearman's rank correlation coefficient r=−0.61). The ${4\text{MC}}^{\text{recal}}$ solutions systematically underpredict the OAR doses as the majority of points are above the line of unity. The maximum DPE in $D_{2\%}$ varies between spinal cord, esophagus, and trachea from 1.54 to 1.78, and the DPE in $V_{20\text{Gy}}$ varies from 0.61 to 1.34 for lung. Of the underpredicted DVH indices, none of them violates their respective dose constrains. The magnitude of DPEs is considerable for only 22 out of the 125 analyzed DVH indices are within ±3% threshold. Of these 22 indices, four are in the GTV and the other 18 are in the OARs.

**Figure 3:**
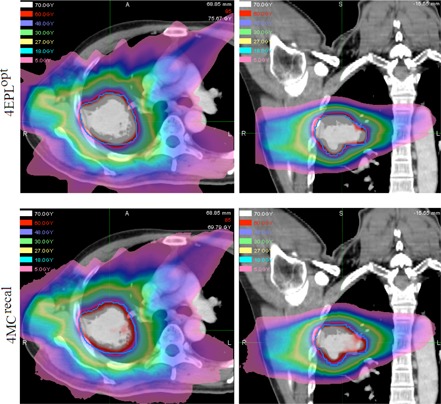
The ${4\text{EPL}}^{\text{opt}}$ and ${4\text{MC}}^{\text{recal}}$ dose distributions are shown in the axial (left column) and coronal (right column) for a 73.8 cc tumor located in the right middle lobe of the lung. The most pronounced difference between these distributions is observed in the prescription dose level of 60 Gy that falls short of covering the planning target volume (outlined by the blue solid line) after ${4\text{EPL}}^{\text{opt}}$ solution was applied to the ${4\text{MC}}^{\text{recal}}$ method.

**Figure 4:**
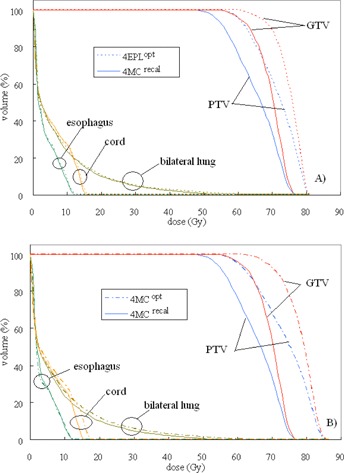
Dose‐volume histograms of the GTV and PTV, and selected critical organs exemplify the two types of errors: dose prediction errors and optimization convergence errors. The dose differences (a) when the ${4\text{EPL}}^{\text{opt}}$ plan undergoes the final dose calculation using two different dose calculation algorithms (EPL and MC); the dose differences (b) when the same final dose calculation algorithm (MC) is used but the dose algorithms used in optimization differ (EPL and MC).

The statistical results are given in Table 2. The first column of the table shows the averaged values of the reference DVH indices over 25 patients. The following column shows the $D_{V}(\text{Gy})$ or $V_{D}(\%)$ intervals at which equivalence tests indicated DPEs are statistically significant. The DPE is statistically significant only in the GTV, as the $D_{98\%}$ interval around the ${4\text{MC}}^{\text{recal}}$ reference value is outside 3% tolerance. For OARs, although the DVH indices are outside the ±3% threshold in 82 out of the 100 DVH indices, the $D_{2\%}$ and $V_{20\text{Gy}}$ intervals around their corresponding reference of ${4\text{MC}}^{\text{opt}}$ averages are within 0%−1.2%. Thus, the DPEs in OARs are clinically insignificant. The ${4\text{EPL}}^{\text{opt}}$ and ${4\text{MC}}^{\text{recal}}$ dose distributions in the axial and coronal planes of a 73.8 cc GTV are shown in Fig. 3. These dose distributions are similar in the low‐to‐medium dose volume, but exhibit observable systematic dose prediction error at the GTV interface with the low‐density lung tissue. The difference between the two types of errors are further exemplified in Fig. 4(a) and 4(b). In Fig. 4(a), the ${4\text{EPL}}^{\text{opt}}$ plan undergoes the final dose calculation using two different dose calculation algorithms (EPL and MC). Consider that MC dose calculation is the gold standard, the ${4\text{EPL}}^{\text{opt}}$ DVHs exhibits a systematic dose prediction error. In Fig. 4(b), the same final dose calculation algorithm was used (MC), but the dose algorithms used in optimization differed. The differences in DVHs between the ${4\text{MC}}^{\text{recal}}$ and ${4\text{MC}}^{\text{opt}}$ solutions demonstrate an optimization convergence error.

**Table 2:** Statistics results of different DVH indices, and the $D_{V}(\text{Gy})$ and $V_{D}(\%)$ intervals at which statistical equivalence test indicate that DPEs (${4\text{EPL}}^{\text{opt}}$ vs. ${4\text{MC}}^{\text{recal}}$) and OCEs (${4\text{MC}}^{\text{recal}}$ vs. ${4\text{MC}}^{\text{opt}}$) are statistically significant (p<0.05) for each DVH index analyzed

	*Average* ${4\text{MC}}^{\text{recal}}\;D_{V}(\text{Gy})$ *or* $V_{D}(\%)$ *used as DPE Reference*	*DPE Interval of* $D_{V}(\text{Gy})$ *or* $V_{D}(\%)$	*Average* ${4\text{MC}}^{\text{recal}}\;D_{V}(\text{Gy})$ *or* $V_{D}(\%)$ *used as OCE Reference*	*OCE Interval of* $D_{v}(\text{Gy})$ *or* $V_{D}(\%)$
${\text{GTV D}}_{98\%}$	6000 cGy	1044 cGy	6821 cGy	1188 cGy
${\text{Cord D}}_{2\%}$	992.7 cGy	7 cGy	1093 cGy	15 cGy
${\text{Trachea D}}_{2\%}$	804 cGy	10 cGy	849 cGy	30 cGy
${\text{Esophagus D}}_{2\%}$	951 cGy	5 cGy	1024 cGy	20 cGy
${\text{Lung V}}_{20\text{Gy}}$	5%	0.0%	6.4%	0.7%

${4\text{MC}}^{\text{recal}}=$ the ray‐tracing optimized 4D plan (${4\text{EPL}}^{\text{opt}}$) recalculated by Monte Carlo; ${4\text{MC}}^{\text{opt}}=$ direct 4D Monte Carlo optimized plan; DPE=dose prediction error; OCE=optimization convergence error; $D_{v}=$ dose received by *V* percentage volume of the OAR; $V_{D}=$ percentage volume receiving at least dose *D*.

**Figure 5:**
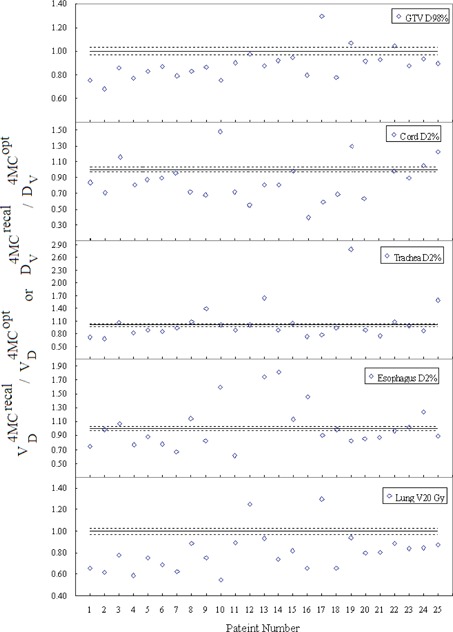
Normalized DVH indices for different organs demonstrating the effects of ${4\text{MC}}^{\text{opt}}$ method. Differences are resulted from the OCEs when ${4\text{MC}}^{\text{recal}}$ and ${4\text{MC}}^{\text{opt}}$ methods are compared. The dotted lines indicate the 3% tolerance and the solid line indicates the baseline when the dose prediction errors are nil.

Results of the per‐patient OCE evaluation are presented in Fig. 5. It can be observed that the OCE plots roughly mirror the corresponding DPE plots. The ${4\text{MC}}^{\text{opt}}$ method corrects both under‐ and overpredicted ${\text{GTV D}}_{98\%}$. For OARs, the majority of normalized DVH indices are smaller than unity, suggesting that the final solutions of the ${4\text{MC}}^{\text{opt}}$ method in sparing the OARs approach to those of the ${4\text{EPL}}^{\text{opt}}$ method. Averages of the normalized $D_{2\%}$ vary from 0.90 for cord, 1.00 for esophagus, and 1.05 for trachea, and the normalized $V_{20\text{Gy}}$ of lung varies from 0.55 to 1.30, with a mean of 0.80. Figure 5 shows that the ${4\text{MC}}^{\text{opt}}$ method reduced cord $D_{2\%}$ to below 18 Gy dose limit in two patients who had their cord $D_{2\%}$ exceeded 18 Gy in the ${4\text{MC}}^{\text{recal}}$ plans. It is noteworthy that there was also one case where the ${4\text{MC}}^{\text{recal}}$ solution yielded cord $D_{2\%}<18\;\text{Gy}$, whereas the ${4\text{MC}}^{\text{opt}}$ method came up with a solution ensuring the PTV but failed to meet the cord dose tolerance. The OCE is within the 3% tolerance level in ten out of the 125 analyzed DVH indices, of which only one is in the GTV. Statistical results of the averaged ${4\text{MC}}^{\text{opt}}$ doses and the corresponding $D_{V}$ or $V_{D}$ intervals are given in the third and fourth columns in Table 2. Since the percentage of $D_{98\%}$ interval in GTV is 17.4%, which is greater than the relative uncertainty level (<5%), the OCE due to the EPL algorithm is definitely significant. Table 2 also demonstrates that the results of statistical tests differ between OARs. Statistical equivalences were observed in the $D_{2\%}$ intervals of cord, trachea, and esophagus within 3.5% of the reference ${4\text{MC}}^{\text{opt}}$ doses. For lung, a statistical equivalence was observed in the $V_{20G}$ interval within 10.4% of the reference ${4\text{MC}}^{\text{opt}}\;V_{20\text{Gy}}$.

**Figure 6:**
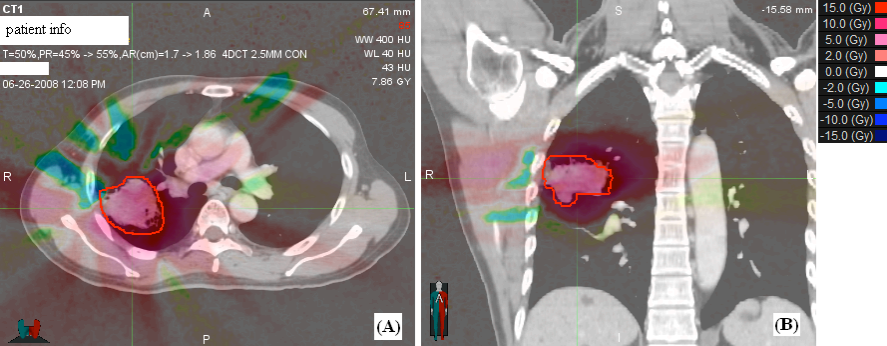
Dose difference maps in the axial (a) and coronal (b) planes for patient 23. They were obtained by subtracting the ${4\text{MC}}^{\text{recal}}$ doses from the ${4\text{MC}}^{\text{opt}}$ doses. The figure the ${4\text{MC}}^{\text{opt}}$ solution manages to attain adequate dose coverage of the PTV medially and posteriorly while it lowers excessive dose in portion of the chest wall.

Figure 6 shows dose difference maps between the ${4\text{MC}}^{\text{recal}}$ and ${4\text{MC}}^{\text{opt}}$ dose distributions. The hot and cold spots seen in the dose difference maps indicate that the ${4\text{MC}}^{\text{opt}}$ solution not only manages to compensate the dose loss caused by out‐scattering electrons not being replaced by in‐scattering electrons in the low‐density lung tissues at the posterior, medial, and craniocaudal side of the GTV, but also lowers doses in the chest wall on the other side. It is also noteworthy that the ${4\text{MC}}^{\text{opt}}$ solution sometimes causes higher normal tissue dose, possibly due to the increased monitor units (MU) required to compensate to electron losses in the low‐density GTV‐to‐PTV regions.

**Figure 7:**
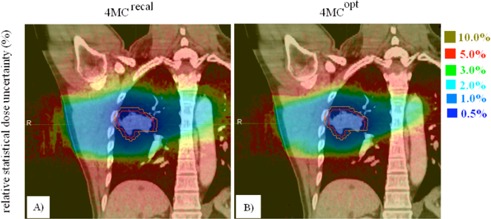
Statistical dose uncertainty between different anatomic regions in the ${4\text{MC}}^{\text{recal}}$ (a) and ${4\text{MC}}^{\text{opt}}$ dose results (b). The red and orange solid lines indicate the gross tumor volume and the planning target volume, respectively.

The relative statistical dose uncertainty maps in Fig. 7(a) and 7(b) highlight the difficulties of evaluating and reporting doses for different structures. The relative dose uncertainties are up to 7% in cord and even higher in the contralateral lung, which receives relatively low doses (Note: relative percentage uncertainty in dose per voxel $\propto \;1/\sqrt{dose}$.^(26)^ This uncertainty is comparable to, and sometimes greater than, the percent $D_{V}$ or $V_{D}$ intervals, thus confounding the results of the statistical equivalence tests.

## IV. DISCUSSION & CONCLUSIONS

The use of 4D CT and deformation image registration made it possible to quantify the effects of DPEs and OCEs due to differences between conventional ray‐tracing and accurate Monte Carlo dose calculation methods in deforming geometry that have not been previously studied in robotic‐based lung SBRT. Our results demonstrated that conventional ray‐tracing correction‐based algorithm failed to account for the lateral electron disequilibrium. As a consequence, the ${4\text{EPL}}^{\text{opt}}$ solution systematically overestimated the ${\text{GTVD}}_{98\%}$ (Fig. 2). Notably, there is systematic clipping of the PTV by the prescription isodose line in the low‐density rind surrounding the GTV (Fig. 3). In this work, a 17.4% dose interval is required to encompass the DPE in ${\text{GTV D}}_{98\%}$, which was considered clinically significant based on the 3% tolerance (Table 1). For the selected OARs, the DPE was found in the range of 3.5% to 8.6%. These results were consistent with other studies that quantified the DPEs in static 3D geometries. For instance, van der Voort van Zyp et al.^(1)^ recalculated 53 patient plans optimized with ray‐tracing algorithm by the same Monte Carlo algorithm as used in this study and reported an averaged decrease of PTV prescription dose coverage by 21% in tumors <3cm and 10% in tumors >5cm. For OARs, they reported DPEs of 7% and 6% in maximum doses of cord and esophagus, respectively.

Through incorporation of MC dose calculation in 4D optimizations, the ${4\text{MC}}^{\text{opt}}$ solutions eliminated the OCEs of the ${4\text{EPL}}^{\text{opt}}$ that caused the PTV to look as if it were covered adequately by the prescription dose, but was indeed seriously underdosed. The ${4\text{MC}}^{\text{opt}}$ solutions ensured adequate PTV coverage, while effectively constrained the GTV dose in excess of that prescribed. This is reflected in the same ${\text{GTV D}}_{98\%}$ interval required to encompass the OCE and the DPE. In Fig. 7, the statistical uncertainty in the GTV was found to be within 5% and was smaller than the 17% dose interval of ${\text{GTV D}}_{98\%}$. Therefore, the OCE was confirmed clinically significant. The OCEs differed between OARs. The percent dose interval $D_{2\%}$ varied from 1.3% for cord to 3.5% for trachea, and for lung within 10.4% of the averaged $V_{20\text{Gy}}$. As shown in the OCE plots (Fig. 5), the ${4\text{MC}}^{\text{opt}}$ solutions result in a slight increase of DVH index values in different OARs. Intuitively, the direct inclusion of OAR constrains in the MC optimization should make available previously unexplored solution space for improvement of the OAR doses. As mentioned in earlier session, the objective parameters were not optimal for individual patients because they were derived from a large number of test cases. When the optimization sequence was such that it always prioritized maximization of the PTV dose coverage and the PTV minimum dose, the solution space available beyond these steps for further optimizations of OARs’ doses became smaller. In addition, doses of the OARs after these steps were in most occasions much less than the clinical objectives defined in the optimization sequence. Thus, the OAR optimization steps were simply skipped without refined objective inputs. For the patient whose cord $D_{2\%}$ exceeded the dose limit 18 Gy in the ${4\text{MC}}^{\text{opt}}$ solution, it is expected that setting tighter maximum dose constraint or reordering the optimization sequence shall reduce the ${\text{cord D}}_{2\%}$ below the dose limit.

Although the OCEs were statistically significant for a few OARs, the percentage of $D_{V}$ and $V_{D}$ intervals were comparable to the statistical dose uncertainty. In fact, statistical fluctuations contributed to noise convergence error of the ${4\text{MC}}^{\text{opt}}$ solution which propagated from one optimization step to the next. The noise convergence error was introduced because the optimization converged to an “optimal” solution of beam weights that was difference from the actual optimal one in a noise‐free dose distribution. In beamlet‐based MC optimization, the noise convergence error was related to the number of simulated particle histories of the MC beamlets and became significant when the statistical uncertainty of the MC beamlets was of the order of 2%.^27^, ^28^ Jeraj and Keall^(28)^ showed that the noise convergence error approximately follows the statistical error. Based on the result of Jeraj and Keall, we expect that the relatively large statistical uncertainty (4%) could cause some deviation of the solution of 4D MCO from the optimal one. We aim to investigate the tradeoff between noise convergence error and computation time in 4D MCO of robotic SBRT in future work.

There have been questions about the reliability of deformation image registration for mapping dose calculated at different breathing geometries to the reference geometry. The effect of applying deformation vectors is to deform voxels of the reference phase image to other individual phase images. In case of large deformation, the remapped undeformed reference dose voxels do not necessarily overlap with the voxels of the dose grids of other breathing phases from which doses are to be remapped. This introduces voxel‐size dependent interpolation error.^(29)^ Furthermore, the deformation image registrations employed in the CyberKnife MultiPlan TPS were based on a simple voxel warping approach without congruent energy and mass mapping. Heath et al.^(30)^ have previously investigated the deformation models defVox and its variation defTetra with and without energy and mass mapping using the VMC++ Monte Carlo code. They suggested that consistent mass and energy mapping methods were essential to get meaningful results in situations where inexact deformation fields were used to provide the geometrical transformation. Nonetheless, the defVox model with energy and mass mapping requires long calculation time. Therefore, the deformation registration model implemented on the MultiPlan TPS may still be considered the most clinically practical approach to estimate the dose in deforming geometries.

## ACKNOWLEDGMENTS

This work is supported by the Hong Kong Adventist Hospital.

## Supporting information

Supplementary Material

## References

[1] van der Voort van Zyp N , Hoogeman M , van de Water S et al. Clinical introduction of Monte Carlo treatment planning: a different prescription dose for non‐small cell lung cancer accoording to tumor location and size. Radiother Oncol. 2010;96(1):55–60. 20430461 10.1016/j.radonc.2010.04.009

[2] Wilcox EE , Daskalov GM , Lincoln H , Shumway RC , Kaplan BM , Colasanto JM . Comparison of planned dose distributions calculated by Monte Carlo and Ray‐Trace algorithms for the treatment of lung yumors with cyberknife: a preliminary study in 33 patients. Int J Radiat Oncol Biol Phys. 2010;77(1):277–84. 20004530 10.1016/j.ijrobp.2009.08.001

[3] Kμnzler T , Fotina I , Stock M , Georg D . Experimental verification of a commercial Monte Carlo‐based dose calculation module for high‐energy photon beams. Phys Med Biol. 2009;54(24):7363–77. 19934489 10.1088/0031-9155/54/24/008

[4] Xiao Y , Papiez L , Paulus R et al. Dosimetric evaluation of heterogeneity corrections for RTOG 0236: Stereotactic body radiotherapy of inoperable stage I‐II non–small‐cell lung cancer. Int J Radiat Oncol Biol Phys. 2009;73(4):1235–42. 19251095 10.1016/j.ijrobp.2008.11.019 PMC2911132

[5] Aarup LR , Nahum AE , Zacharatou C et al. The effect of different lung densities on the accuracy of various radiotherapy dose calculation methods: Implications for tumour coverage. Radiother Oncol. 2009;91(3):405–14. 19297051 10.1016/j.radonc.2009.01.008

[6] Bergman AM , Bush K , Milette M‐P , Popescu IA , Otto K , Duzenli C . Direct aperture optimization for IMRT using Monte Carlo generated beamlets. Med Phys. 2006;33(10):3666–79. 17089832 10.1118/1.2336509

[7] Jin L , Wang L , Li J , Luo W , Feigenberg SJ , Ma CM . Investigation of optimal beam margins for stereotactic radiotherapy of lung‐cancer using Monte Carlo dose calculations. Phys Med Biol. 2007;52(12):3549–61. 17664559 10.1088/0031-9155/52/12/014

[8] Starkschall G , Britton K , McAleer MF et al. Potential dosimetric benefits of four‐dimensional radiation treatment planning. Int J Radiat Oncol Biol Phys. 2009;73(5):1560–65. 19231098 10.1016/j.ijrobp.2008.12.024

[9] Söhn M , Weinmann M , Alber M . Intensity‐modulated radiotherapy optimization in a quasi‐periodically deforming patient model. Int J Radiat Oncol Biol Phys. 2009;75(3):906–14. 19747782 10.1016/j.ijrobp.2009.04.016

[10] Heath E , Unkelbach J , Oelfke U . Incorporating uncertainties in respiratory motion into 4D treatment plan optimization. Med Phys. 2009;36(7):3059–71. 19673205 10.1118/1.3148582

[11] Lee L , Ma Y , Ye Y , Xing L . Conceptual formulation on four‐dimensional inverse planning for intensity modulated radiation therapy. Phys Med Biol. 2009;54(13):N255–N266. 19521008 10.1088/0031-9155/54/13/N01 PMC12121644

[12] Chan MKH , Kwong DLW , Ng SCY , Tam EK , Tong AS . Investigation of four‐dimensional (4D) Monte Carlo dose calculation in real‐time tumor tracking stereotatic body radiotherapy for lung cancers. Med Phys. 2012;39(9):5479–87. 22957615 10.1118/1.4739249

[13] Kilby W , Dooley JR , Kuduvalli G , Sayeh S , Maurer CR Jr . The CyberKnife$rg Robotic Radiosurgery System in 2010. Technol Cancer Res Treat. 2010;9(5):431–38. 20815415 10.1177/153303461000900502

[14] Sharma SC , Ott JT , Williams JB , Dickow D . Clinical implications of adopting Monte Carlo treatment planning for CyberKnife. J Appl Clin Med Phys. 2010;11(1):3142. 20160699 10.1120/jacmp.v11i1.3142 PMC5719782

[15] Wilcox EE and Daskalov GM . Accuracy of dose measurements and calculations within and beyond heterogeneous tissues for 6MV photon fields smaller than 4cm produced by Cyberknife. Med Phys. 2008;35(6):2259–66. 18649456 10.1118/1.2912179

[16] Ma CM , Li JS , Deng J , Fan J . Implementation of Monte Carlo dose calculation for CyberKnife treatment planning. J Phys Conf Ser. 2008;102(1).

[17] Ma CM and Li J . Dose specification for radiation therapy: dose to water or dose to medium? Phys Med Biol. 2011;56(10):3073–89. 21508447 10.1088/0031-9155/56/10/012

[18] West J , Maurer CJ , Dooley J , Rohde GK . Deformable registration of abdominal CT iamges: tissue stiffness constraints using B‐Splines. SPIE Medical Imaging 2006. SPIE 2006;6144.

[19] West J , Maurer C , Dooley J . 4D Planning: dose calculation that accounts for moving beams and tissue deformation [abstract]. Med Phys. 2008;35(6):2841.

[20] Stancanello J , Berna E , Cavedon C et al. Preliminary study on the use of nonrigid registration for thoracoabdominal radiosurgery. Med Phys. 2005;32(12):3777–85. 16475777 10.1118/1.2103428

[21] Schlaefer A and Schweikard A . Stepwise multi‐criteria optimization for robotic radiosurgery. Med Phys. 2008;35(5):2094. 18561685 10.1118/1.2900716

[22] ICRU. Prescribing, recording, and reporting intensity‐modulated radiation therapy (IMRT). ICRU Report 83. Bethseda, MD: ICRU; 2010.

[23] Sakthi N , Keall P , Mihaylov I et al. Monte Carlo–based dosimetry of head‐and‐neck patients treated with SIB‐IMRT. Int J Radiat Oncol Biol Phys. 2006;64(3):968–77. 16458782 10.1016/j.ijrobp.2005.09.049

[24] Papanikolaou N , Battista JJ , Boyer AL et al. Tissue inhomogeneity corrections for megavoltage photon beams. AAPM Report No. 85. Report of Task Group No. 65 of the Radiation Therapy Committee of the American Association of Physicists in Medicine. Madison, WI: Medical Physics Publishing; 2004.

[25] Mihaylov IB and Siebers JV . Evaluation of dose prediction errors and optimization convergence errors of deliverable‐based head‐and‐neck IMRT plans computed with a superposition/convolution dose algorithm. Med Phys. 2008;35(8):3722–27. 18777931 10.1118/1.2956710 PMC2673652

[26] Chetty IJ , Rosu M , Kessler ML et al. Reporting and analyzing statistical uncertainties in Monte Carlo–based treatment planning. Int J Radiat Oncol Biol Phys. 2006;65(4):1249–59. 16798417 10.1016/j.ijrobp.2006.03.039

[27] Ma CM , Li JS , Jiang SB et al. Effect of statistical uncertainties on Monte Carlo treatment planning. Phys Med Biol. 2005;50(5):891–907. 15798263 10.1088/0031-9155/50/5/013

[28] Jeraj R and Keall P . The effect of statistical uncertainty on inverse treatment planning based on Monte Carlo dose calculation. Phys Med Biol. 2000;45(12):3601–13. 11131187 10.1088/0031-9155/45/12/307

[29] Rosu M , Chetty IJ , Balter JM , Kessler ML , McShan DL , Ten Haken RK . Dose reconstruction in deforming lung anatomy: dose grid size effects and clinical implications. Med Phys. 2005;32(8):2487–95. 16193778 10.1118/1.1949749

[30] Heath E , Tessier F , Kawrakow I . Investigation of voxel warping and energy mapping approaches for fast 4D Monte Carlo dose calculations in deformed geometries using VMC++. Phys Med Biol. 2011;56(16):5187–202. 21791733 10.1088/0031-9155/56/16/007

