# Supplementary figures and images for: Evaluation of dose prediction error and optimization convergence error in four‐dimensional inverse planning of robotic stereotactic lung radiotherapy
Source: J Appl Clin Med Phys. 2013 Jul 8;14(4):182–95. doi: 10.1120/jacmp.v14i4.4270 (PMC5714544; doi:10.1120/jacmp.v14i4.4270)

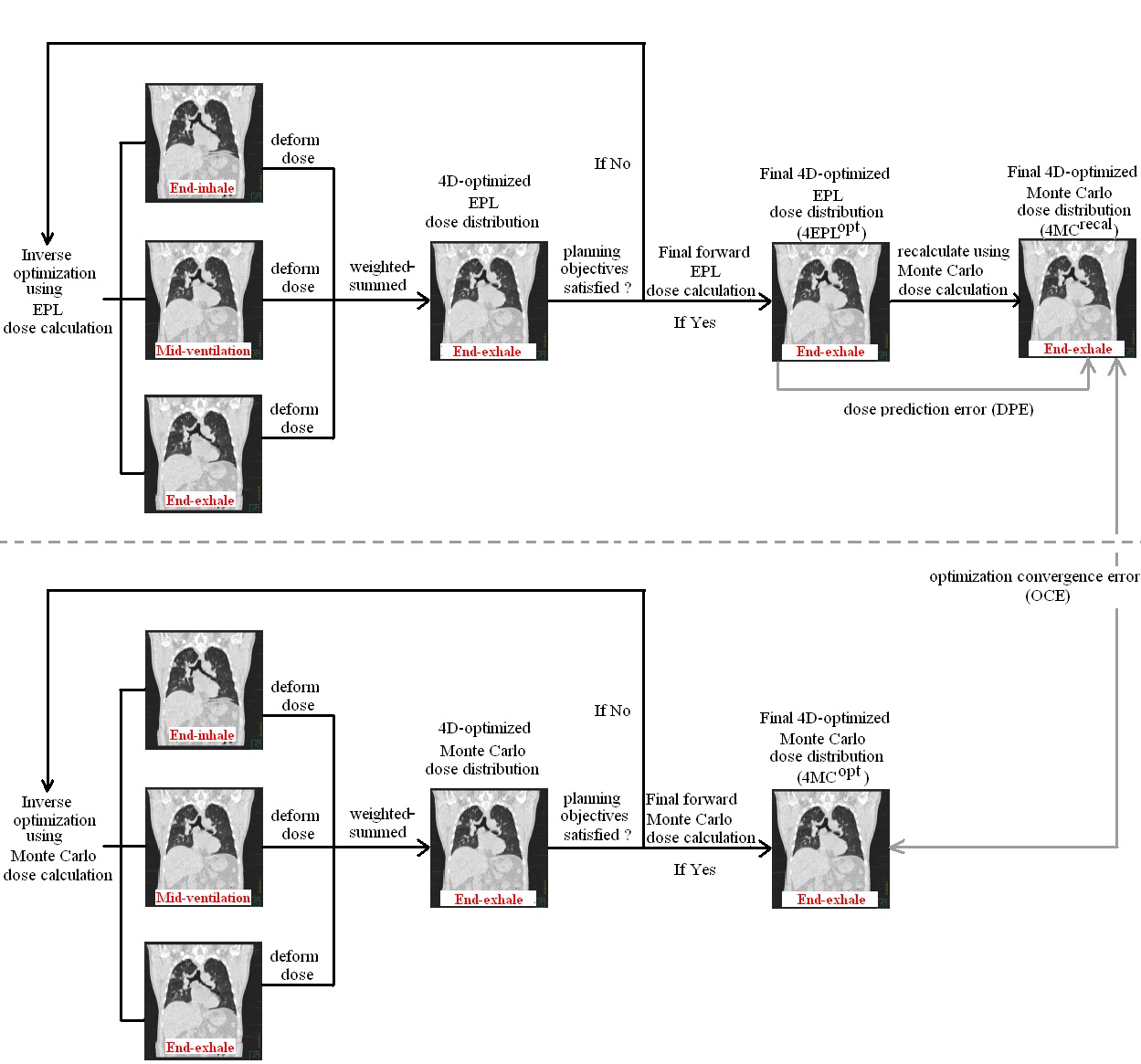

Supplement: Supplementary file 1 — Supplementary Material [file ACM2-14-182-s001.jpg]
